# Baicalin-Loaded Lipid–Polymer Hybrid Nanoparticles Inhibiting the Proliferation of Human Colon Cancer: Pharmacokinetics and In Vivo Evaluation

**DOI:** 10.3390/polym15030598

**Published:** 2023-01-24

**Authors:** Yassine Riadi, Obaid Afzal, Mohammed H. Geesi, Waleed H. Almalki, Tanuja Singh

**Affiliations:** 1 Department of Pharmaceutical Chemistry, College of Pharmacy, Prince Sattam Bin Abdulaziz University, Al-Kharj 11942, Saudi Arabia; 2 Department of Chemistry, College of Science and Humanities in Al-Kharj, Prince Sattam Bin Abdulaziz University, Al-Kharj 11942, Saudi Arabia; 3 Department of Pharmacology and Toxicology, College of Pharmacy, Umm Al-Qura University, Mecca 24382, Saudi Arabia; 4 University Department of Botany, Patliputra University, Patna 800020, India

**Keywords:** baicalin, natural products, lipid–polymer hybrid nanoparticles, colon cancer, particle size, in vitro drug release, pharmacokinetics, bio-distribution

## Abstract

This research work is focused on pharmacokinetic and biochemical experiments to assess baicalin-loaded lipid–polymer hybrid nanoparticles (LPHNPs) with colon-targeting specificity. The nanoprecipitation method was used to develop the LPHNPs, and the characterized formulation revealed the 184.3 nm particle size, PDI of 0.177, spherical shape, and zeta potential of −19.8 mV. The baicalin LPHNPs are said to be poorly absorbed in the stomach and small intestine, and in vitro drug release tests have shown that the drug is released mostly in the caecal fluid. Additionally, the LPHNPs showed stability and nonsignificant drug loss at 25 °C for 3 months. The least viable population of baicalin-loaded LPHNPs was detected at a lower IC_50_ value after 48 h, and no cytotoxicity was observed by blank suspension and blank LPHNPs up to the concentration of 100 µg/mL. Apart from this, the pharmacokinetics study showed that baicalin from LPHNPs is much less absorbed and least available in the blood plasma and maximum available in the colon. Concurrently, organ distribution studies demonstrated that baicalin-loaded LPHNPs were distributed more widely in the colon compared to baicalin suspension. Moreover, baicalin-loaded LPHNPs were found to be superior to a baicalin suspension in reducing elevated liver enzyme levels. In a nutshell, baicalin-loaded LPHNPs show superior efficacy and can be maximally localized into the colon rectal cancer along with systemic availability of the drug.

## 1. Introduction

Colon cancer (colorectal cancer or CRC) is the second leading cause of cancer-related mortality in women and men worldwide. There are still an estimated 100,000 new cases and 50,000 deaths annually, making it the third greatest cause of cancer mortality [1,2]. However, developing new and effective treatments for colon cancer remains a major challenge. Since conventional chemotherapeutic treatments are fraught with cytotoxicity and drug resistance, researchers have turned their attention to plant-based natural chemicals [3,4]. A number of promising medications have been produced and tested in clinical trials based on natural products for a wide range of illnesses, including cancer [5,6]. These natural chemicals can give new targets for therapeutic cancer treatment if we better grasp their biological activities and mechanisms [5,6]. 

Flavonoids are naturally occurring polyphenols found in many fruits and vegetables, as they have been linked to a variety of health benefits and roles in physiological and pathological processes in humans [7]. The plant *Scutellaria baicalensis Georgi* contains the phenolic flavonoid compound baicalin (5,6,7-trihydroxyflavone), which has been used extensively in traditional Chinese herbal medicine to treat many illnesses, including infections and inflammations [8]. The current literature reported that the daily intake of baicalin at the dose of 30 mg/kg causes significant inhibition of HCT-116 tumor growth at the end of 4 weeks. The in vivo anti-tumor studies of baicalin also supported in vitro anticancer effects [9]. However, poor water solubility and restricted bioavailability limits its possible use in CRC [9].

Chitosan (CS), the only naturally occurring positive-charge polysaccharide, offers biodegradability, biocompatibility, nontoxicity, etc. [10]. An amine group is bonded to carbon 2 in glucose in accordance with the chemical structure of CS, making the compound a polycation in acidic environments and capable of exhibiting mucoadhesive properties when it comes into contact with the mucosa because of the presence of negatively charged sialic acids [11]. Due to their mucoadhesive properties and capacity to increase permeability, CS nanoparticles are also well-suited for local distribution at the colon sites and other mucosal regions [11]. Curcumin nanoparticles loaded with CS biopolymer and bovine serum albumin were created by Rajan et al. They noticed that increasing concentration made the selective drug targeting of colorectal cancer cells more successful [12]. CS-coated nanoparticles (NPs) were used to target tumors and increase the anticancer activity. Intraperitoneally injected CS-PLGA-TA-E dramatically reduced tumor number and volume and normalized colon histology in colon cancer patients [13]. 

To overcome the limitations of individual liposomes and polymeric nanoparticles (NPs), traditional lipid- and polymer-based nanoparticles have many drawbacks, including dose-related toxicity, poor selectivity, fast diffusion systemically, and short half-lives in the circulation [14,15]. To improve drug accumulation in the colon, lipid–polymer hybrid nanoparticles (LPHNPs) can be employed to circumvent the physiological and anatomical hurdles. By wisely controlling the particle size around 0.23 µm, CRC may be targeted passively [14]. LPHNPs have emerged as a viable drug delivery technology. LPHNPs encompass the benefits of both liposomes and nanoparticles, while overcoming their drawbacks [14]. Instead, a well-constructed hybrid nanoparticle will have a hydrophobic polymeric core to slow the release of water-soluble drugs and a biocompatible lipid shell to protect the core from the stoma environment [14,15]. Biocompatible, biodegradable, prolonged drug-release pattern, and enhanced loading-capacity-tailored drug delivery vehicles can improve chemotherapeutic effectiveness against specific cell types [14,15]. To address this challenge, baicalin can be loaded into LPHNPs and administered in a regulated and effective fashion. Therefore, the aim and purpose of these experimental studies were to develop and assess LPHNPs for baicalin administration that may be used to selectively target CRC.

## 2. Materials and Methods

### 2.1. Chemicals and Reagents 

Baicalin was procured from Sigma Aldrich (United States). 1,2-distearoyl-sn-glycero-3-phosphoethanolamine-*N*-[amino(polyethyleneglycol)-2000 (DSPE-PEG2000) was obtained from Lipoid GmbH (Ludwigshafen, Germany). Chitosan, soya lecithin, 1,2-distearoyl-sn-glycero-3-phosphoethanolamine-*N*-[amino(polyethyleneglycol)-2000 (D70SPE-PEG2000), DMSO, ethanol, and phosphate-buffered saline (PBS; pH 7.4) were procured from Merck (Bengaluru, India) and Qualigens fine chemicals (Mumbai, India). Poly(lacticglycolic)acid was received as gift sample from Eonik India Pvt. Ltd. (Mumbai, India). Poloxamers and Tween 80 were purchased from Fischer Scientific Pvt. Ltd. (Mumbai, India). The solvents used were of HPLC grade. All the reagents and solvents used were of high analytical consistency and obtained from authorized manufacturers. 

### 2.2. Preparation of Baicalin-Loaded LPHNPs

The nanoprecipitation method was used to develop LPHNPs [14,15,16]. In a chitosan–ethanol solution, 20 mg of baicalin were dissolved at a concentration of 3 mg/mL. Soya lecithin and 0.5 mg of D70SPE-PEG2000 (D70SPE-PEG2000) were then added in a 5% *w*/*v* DMSO-containing aqueous solution that was preheated to 70 °C, and the mixture was stirred. The chitosan–ethanol solution was then added to the warmed lipidic aqueous solution at a 1 mL/min rate, and vortexed for five minutes after each addition. Afterward, the solution was kept at room temperature for three hours and stirred. Unbound drug molecules and any leftover organic solvent were removed by dialysis against PBS (pH 7.4) in the presence of an organic solvent in a dialysis tube (Spectra/Por 6 membrane, MWCO 1000; Spectrum Labs, Cincinnati, OH, USA) that had been previously used to remove the drug. Additionally, mannitol (3% *w*/*w*) was added to the nanoparticles to serve as a cryoprotectant before they were freeze-dried to give a fine powder.

### 2.3. Characterization of Baicalin-Loaded LPHNPs

#### 2.3.1. Particle Size, Polydispersity Index, Zeta Potential, and Transmission Electron Microscopy

A number of parameters, including particle size, zeta potential, surface morphology, drug entrapment efficacy (EE), and loading capacity (LC) [16], were evaluated for baicalin-loaded LPHNPs. Electrophoretic mobility under an electric field (Nano ZS, Malvern, UK) was utilized to quantify the zeta potential, whereas dynamic light scattering (DLS) was employed to determine NPs size [16]. Transmission electron microscopy (TEM) was used to characterize the surface morphology of the baicalin-loaded LPHNPs [14,15]. When applied to a membrane-coated grid surface, the LPHNPs solution was diluted and dyed with a drop of 1% phosphor tungstic acid (PTA). The excess fluid was removed, and the grid was allowed to dry for one minute (HR-TEM; Fei, Electron Optics) and was studied using a high-resolution transmission electron microscope.

#### 2.3.2. Encapsulation Efficiency (Ee) and Drug Loading Capacity (Lc)

LPHNPs dispersion was processed by Amicon centrifugal filters to evaluate the entrapment efficiency (EE) (Ultracel-0.5, 100 kDa, Milipore Corp., Mumbai, India) [14,16]. This filtrate was analyzed to see how much drug was left unentrapped. The actual amount of drug encapsulated in the LPHNPs was evaluated by centrifuging a nanoparticle dispersion at high speed (15,000 rpm for 15 min) to obtain the pellet and then re-dispersing it in 5 mL ethanol for lysis to determine the amount of drug encapsulated in LPHNPs [16]. To do the HPLC (Shimadzu LC-2010CHT) analysis, the mixture was filtered and diluted to the proper concentration. In this study, a reverse-phase C_18_ column (10 µ, 4.6 mm by 250 mm) was used with a mobile phase involving phosphate buffer saline (pH 7.4) and acetonitrile (*v*/*v*) at a ratio of 3:1. The sample was examined at a wavelength of 460 nm with a flow rate of 1 mL/min. The following is an equation that may be used to determine encapsulation efficiency and drug loading capacity.

(1) $$EE\;\left(\%\right)=\frac{\;Total\;quantity\;of\;\mathrm{Baicalin}-A-Unbound\;\mathrm{Baicalin}\;}{Tota\;weight\;of\;\mathrm{Baicalin}\;added}\times 100$$

(2) $$LC\;\left(\%\right)=\frac{Total\;amount\;of\;\mathrm{Baicalin}\;encapsulated\;in\;LPHNPs}{Total\;amount\;of\;LPHNPs\;\;weight}\times 100\;$$

### 2.4. In Vitro Drug Release

In vitro drug-release experiments were conducted using the dialysis bag method in 0.1 M HCL (2 h) phosphate buffer of pH 7.4 (3 h) to determine the ability of LPHNPs to tolerate the acidic stomach and basic intestinal environments [14,17]. Briefly, a fixed quantity of baicalin-loaded LPHNPs that is equivalent to 20 mg of the drug was dispersed in 2.5 mL of 0.1 M HCL (2 h) and a phosphate buffer of pH 7.4 (3 h) separately into a dialysis bag. For a further 15 min, the dialysis bag was submerged in a solution containing 25 mL of the release medium. Sink conditions were maintained by periodically removing samples and replacing them with fresh media. Meanwhile, the drug-release studies in rat cecal content medium continued to assess the baicalin-loaded LPHNPs′ potential to release baicalin in a physiological setting. The dialysis bag was dipped into a container containing 25 mL of the release medium, and then 2.5 mL of fresh rat cecal content at a concentration of 5% was added. This experiment was conducted with a constant supply of carbon dioxide, since the cecum is anaerobically adapted. The experiment was run for 19 h, which is similar to the typical colon transit duration of 20–30 h, by removing 0.5 mL of the sample at regular intervals and replacing it with 0.5 mL of fresh 5% rat cecal content bubbled with CO_2_. High-performance liquid chromatography (HPLC) was utilized to identify baicalin in the withdrawn samples after they were filtered via a 0.22 µm syringe filter. In contrast, formulations containing cecal material were excluded from the control research. Each case of drug release testing was conducted in triplicate.

### 2.5. Stability Studies

Baicalin-loaded LPHNPs were tested in two different environments: 25 °C/60% RH and 40 °C/75% RH [14,16]. Glass vials with rubber stoppers and aluminum seals crimped on top were used to store the lyophilized product. Stability tests were performed on samples taken at 0, 1, 2, 4, 8, and 12 weeks (3 months). Following the time given in the methods, HPLC analysis of the PS, PDI, EE, and LC formulations was conducted on the samples.

#### Drug Content

The amount of drug present in the developed systems was evaluated by extracting the drug in ethanol at regular intervals and then evaluating it with a HPLC analysis as per the method reported in Section 2.3.2.

### 2.6. Cell Culture and Cell Viability Assay 

A cell viability assay was performed by the standard cell-counting kit-8 (CCK-8) method. HCT-116 cells were seeded into cell culture media at a density of 1 × 10^4^ cells per well in a 96-well plate [18]. The plates were incubated for a further 24–48 h after 12 h, at which point 100 µL of the medium comprising 0–100 µg/mL of baicalin was added. After two washes with PBS, the cells were kept on a plate with 100 µL of new media containing 10 µL of CCK-8 solution for an additional 3 h. After washing the medium, the absorbance was measured with a microplate reader (TECAN M200 infinite Pro) at 460 nm. The indicated error bars are representative of the normal variability between repeats; all experiments were done in triplicate. The optical density (OD) recorded was used in the following equation to calculate the percentage of viable cells.

(3) $$Cell\;viability\;\left(\%\right)=\;\frac{\left(OD\;of\;treated\;cells-OD\;of\;blank\;media\right)}{\left(OD\;of\;control\;cells-OD\;of\;blank\right)}\;\times \;100$$

### 2.7. Animal Studies: Pharmacokinetic and Organ Distribution Studies

The in vivo study was conducted with acquired permission (1840/PO/ReBi/S/15/CPCSEA) as per the Institutional Animal Ethics Committee (IAEC) guidelines, at Patliputra University (Patna, India). Furthermore, experimental studies involving animals were performed in accordance with IAEC requirements. The male albino rats (200–230 g) were housed in polypropylene cages and provided with a regular laboratory diet (Lipton feed, Mumbai, India) and water ad libitum in a controlled environment (25 ± 2 °C, 55 ± 5% relative humidity). The first group served as the "control" in this experiment. The second group received the baicalin suspension (equivalent to 10 mg of the drug), whereas the third group of rats received baicalin-loaded LPHNPs containing an equivalent amount of the drug. Oral gavages were used to feed the suspension form, which was then followed by a large amount of water consumption.

#### 2.7.1. In Vivo Drug-Absorption Study

Samples of rat blood (1 mL each) were taken from their tail veins in vacutainer tubes at 0, 0.5, 1, 2, 3, 6, 12, and 24 h intervals for the control and tests (first, second, and third groups). We were able to split the plasma by adding 2.0 mL of the mobile phase to 0.5 mL of plasma and spinning the mixture at 10,000 rpm for 15 min. The supernatant was collected and dried to dryness, and the residue was reconstituted with the mobile phase. The drugs were analyzed using high-performance liquid chromatography (HPLC). Non-compartmental analysis, also known as model independent analysis, was used to determine the pharmacokinetic (PK) parameters with WinNonLin version 4.0. (Pharsight Corp., Mountain View, CA, USA). Plasma concentration versus time profiles were used to calculate the highest plasma concentration (C_max_) and the time at which it occurred (t_max_). To determine the area under the concentration-time curve (AUC_0–t_), the linear trapezoidal technique was used.

#### 2.7.2. Organ Distribution Study 

Whole GITs from the rats of the second and third groups were removed at 0, 2, 4, 6, and 8 h. The mesenteric and fatty tissues were then isolated [17]. The gastrointestinal tract (GIT) was further subdivided into the stomach, the small intestine, the caecum, and the colon. A micro-tissue homogenizer (Remi Ltd., Mumbai, India) was used to homogenize these organs with 25 mL of phosphate buffer pH 7.4, and then 1.5 mL of acetonitrile was added and left to settle for 60 min. HPLC analysis was used to determine the concentration of the drug by diluting the supernatants with the mobile phase.

### 2.8. Biochemical Investigation

A blood sample (5 mL) was obtained through a cardiac puncture following the animal′s death, and the chest was opened via bilateral thoracotomy. For the purpose of calculating white blood cell and platelet counts, 2 mL of blood were treated with sodium EDTA. The residual blood was centrifuged at 3000 rpm for 15 min to separate the serum. The serum was then stored at −20 °C until biochemical analysis was performed. A cell counter (Sysmex-K100, Transasia Biomedicals Ltd., Mumbai, India) was used to count white blood cells and platelets. The levels of SGOT and SGPT, alkaline phosphatase, creatine, and albumin were determined using the UV kinetic methods, viz. the p-nitrophenyl phosphate (PNPP), alkaline picric acid, and BCG (bromocresol green) methods [19]. Blood levels of SGOT, SGPT, albumin, creatinine, and alkaline phosphatase were determined using a clinical chemistry analyzer (ERBA Chem-5 Plus Transasia Biomedicals Ltd., Mumbai, India).

### 2.9. Statistical Analysis

Analysis of variance (ANOVA) was used to analyze the statistical data. The Student–Newman–Keuls multiple comparison test was used to establish the statistical significance of the differences. *p* values less than 0.05 were considered significant in every case. 

## 3. Results

### 3.1. Preparation of Baicalin-Loaded LPHNPs

Baicalin-loaded LPHNPs were developed by the nanoprecipitation method. As a polymer, PLGA was added to DMSO, tripalmitin and, 1, 2-distearoyl-sn-glycero-3-phosphoethanolamine-*N*-[amino(polyethyleneglycol)-2000 (D70SPE-PEG2000) in 5% w/v methanol for complete solubilization. Dropwise addition of PLGA-DMSO was made into the preheated lipidic solution, followed by gentle agitation for 2 h at 30 °C, resulting in LPHNPs. For developing the drug-loaded LPHNPs, baicalin was added in polymeric solution and the same above-mentioned process was employed. All of the components of the hybrid nanoparticles were also shown to be biodegradable and nontoxic in clinical settings. The polymeric core contains the baicalin, and the functionalized lipids form self-assembled shells surrounding the core. In addition, the developed formulations were put through a series of characterization investigations, which are detailed below. 

### 3.2. Characterization of Baicalin-Loaded LPHNPs: Particle Size Distribution, Zeta Potential, Transmission Electron Microscopy, and Entrapment Efficiency (%ee)

**Figure 1:**
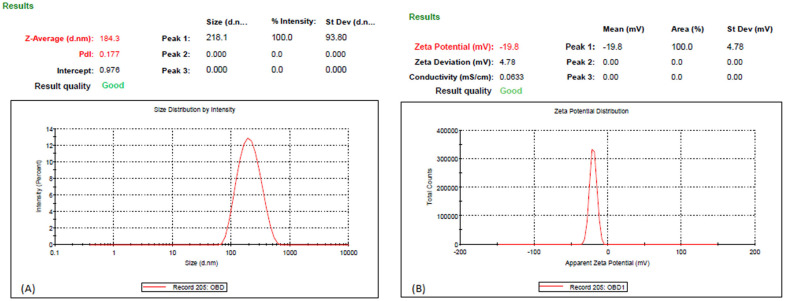

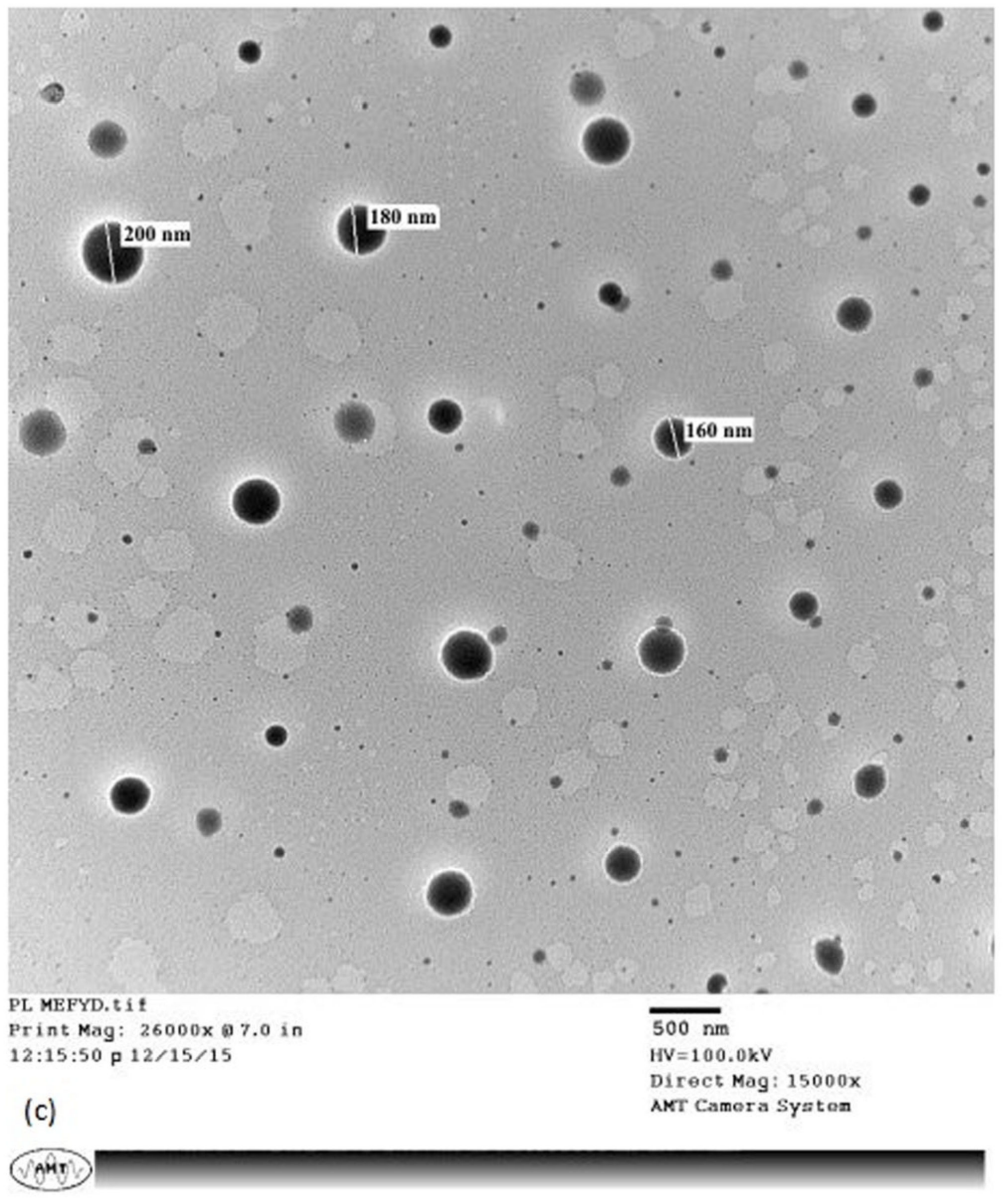
(**A**,**B**) Particle size of baicalin-loaded LPHNPs and zeta potential, respectively. (**C**) Electron transmission microscopy (TEM) of baicalin-loaded LPHNPs.

The developed baicalin-loaded LPHNPs are depicted in Figure 1A, and they exhibit a relatively uniform particle size distribution (PDI = 0.177) and a particle size of 184.3 nm at the 95 % confidence interval. Furthermore, these results confirmed the nanometer scale of LPHNPs with homogeneous dispersion. Moreover, these results are supported by the previous published literature [14]. As reported in the literature, the administration of nanoparticles below size 200 nm leads to significantly higher accumulation in colon cancer via passive targeting [14]. The experimental results showed a particle size of 184.3 ± 8.5 nm and the size-dependent passive targeting of baicalin-loaded LPHNPs to the colon cancer cells was further confirmed in the biodistribution study section given below. The zeta potential exists at −19.8 mV (Figure 1B). This zeta value showed stable and non-aggregated formulation. The zeta potential is the electrostatic potential produced by the liquid layer that is linked to the dispersed particle and the dispersing medium [20]. The physical stability of the formulation is improved, and the repulsion between the nanoparticles is increased because of the negative charge on the formulation. In addition, the fatty acids released during the slow hydrolysis of tripalmitin may be responsible for the negative charge [14,20]. Figure 1C depicts a TEM photomicrograph of baicalin-loaded LPHNPs, which were between 160 and 200 nm in size. Additionally, this variance in the value of TEM as compared to the DLS method was observed [21]. This occurs as a result of the formulation size being established by TEM in the solid state, while the hydrodynamic diameter is estimated by a zeta sizer in the fluid state [21,22]. Additionally, the TEM pictures revealed a spherical surface of the aforementioned formulation with a uniform particle size distribution, and the result was almost confirmed by the DLS measurements [21,22]. Additionally, TEM images of the same formulations are displayed in Figure 1C, and this image demonstrates a further notable distinction between several tiny, dense, white spherical forms that are coated in lipids and may be designated as LPHNPs. Our findings are consistent with previous published articles, and our findings also validated their findings [14,21,22]. The entrapment efficiency (%EE) of the formulation was found to be 90.12 ± 11.77%. Apart from this, the effectiveness of the preparation method was also reflected by the quality of LPHNPs.

### 3.3. In Vitro Drug Release Studies

**Figure 2:**
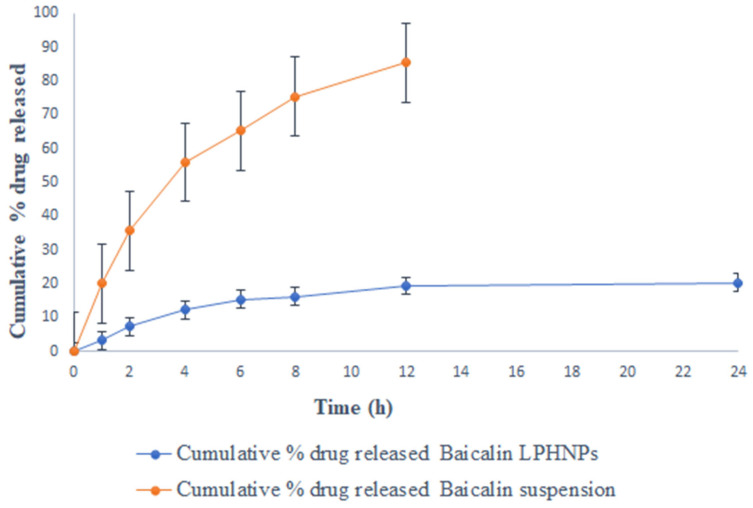
Percentage of in vitro release of baicalin-loaded LPHNPs in simulated gastric fluid (pH 1.2). Data expressed as mean ± SD (n = 6).

**Figure 3:**
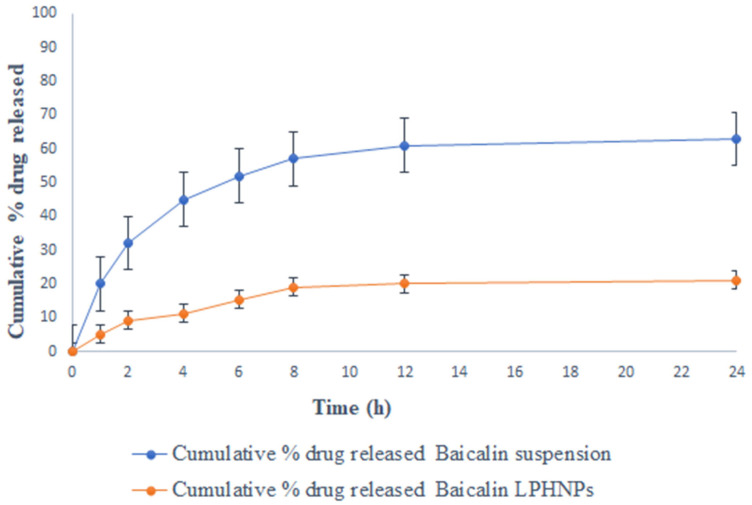
Percentage of in vitro release of baicalin-loaded LPHNPs and baicalin suspension at pH 7.4. Data expresses as mean ± SD (n = 6).

**Figure 4:**
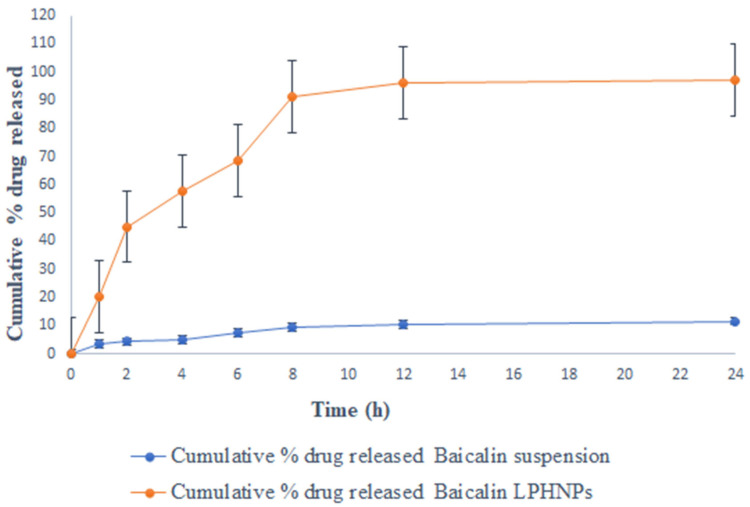
Percentage of in vitro release of baicalin-loaded LPHNPs and baicalin suspension in rat cecal content. Data expressed as mean ± SD (n = 6). Statistical analysis indicated a very significant difference (*p* < 0.001).

Figure 2 shows the drug release performance of baicalin suspension and baicalin-loaded LPHNPs in three distinct media (0.1-N HCl, phosphate buffer pH 7.4, and rat cecal content) at regular intervals of time. The findings show that the baicalin suspension and baicalin-loaded LPHNPs release the drug into the stomach and intestine. Baicalin-loaded LPHNPs and baicalin suspension released only 16.22 ± 1.21% and 75.27 ± 3.33% of the drug over the course of 8h in simulated gastric fluid (pH 1.2), respectively (shown in Figure 2). Furthermore, at the end of 24 h of study, baicalin-loaded LPHNPs and baicalin suspension were observed 20.24 ± 1.31% and 85.45 ± 5.22%, respectively. While at pH 7.4, for 8 h baicalin-loaded LPHNPs and baicalin suspension released their drug 19.04 ± 1.56% and 57 ± 3.21%, respectively (Figure 3), whereas at the end of 24 h, baicalin-loaded LPHNPs and baicalin suspension found drug release 21.04 ± 2.41% and 63 ± 6.43%, respectively. Therefore, from this study, it was concluded that a much less amount of Baicalin is released from the formulation at the extended period. The ideal oral drug-delivery system containing baicalin-loaded LPHNPs over baicalin suspension would release less baicalin in the physiological milieu of the stomach and small intestine (Figure 2 and Figure 3). Baicalin-loaded LPHNPs would be perfect for an oral drug-delivery system since they would not release their active ingredient higher in the acidic and basic conditions of the stomach and small intestine [23]. Baicalin-loaded LPHNPs and baicalin suspension with rat cecal content had a percentage release of 91.24 ± 10.11% and 9.33 ± 1.05%, respectively, at the end of 8 h (shown in Figure 4), whereas at the end of 24 h, for baicalin-loaded LPHNPs and baicalin suspension, their drug released at 97.24 ± 8.11% and 11.56 ± 1.21%, respectively. Statistical analysis indicated a very significant difference (*p* < 0.001). This work shows that microbial degradation of the lipids and polymers inside LPHNPs is responsible for the release of baicalin in the physiological milieu of the colon [17].

### 3.4. Stability Studies 

Table 1 summarizes the results of stability testing of baicalin-loaded LPHNPs at 25 °C and 60% RH and stress at 40 °C and 75% RH. The differences in particle size, PDI, and entrapment efficiency indicated in Table 1 were not statistically significant at 25 °C and 60% RH. At 40 °C and 75% RH, where the formulation′s kinetic energy is increased and leads to an increase in the particle size collisions, accumulation and growth in particle size were observed [14,21,22]. The results show that a formulation kept at 25 °C and 60% RH for 3 months has a monomodal particle size distribution and shows no statistically significant changes in PDI and EE. This observation is also consistent with findings of the stability of LPHNPs that have been reported in the scientific literature [21,22].

#### Drug Content

The modified formulation′s stability trials in terms of drug content (Table 2) were carried out at 25 °C/60% RH and 40 °C/75% RH for at least 12 weeks. After all, the specified week in Table 2, the percentage drug content was calculated as given in Section 2.3.2. The optimized LPHNPs were shown to be stable for six weeks at all 25 °C/60% RH, but the LPHNPs remained stable at 25 °C (for 12 weeks) and at 40 °C drug loss was significant. In these findings, it is shown that the improved LPHNPs may be maintained at 25 °C/60% RH, without needing to be refrigerated.

### 3.5. Baicalin Inhibits Cell Growth of Hct-116 Colon Cancer Cells

**Figure 5:**
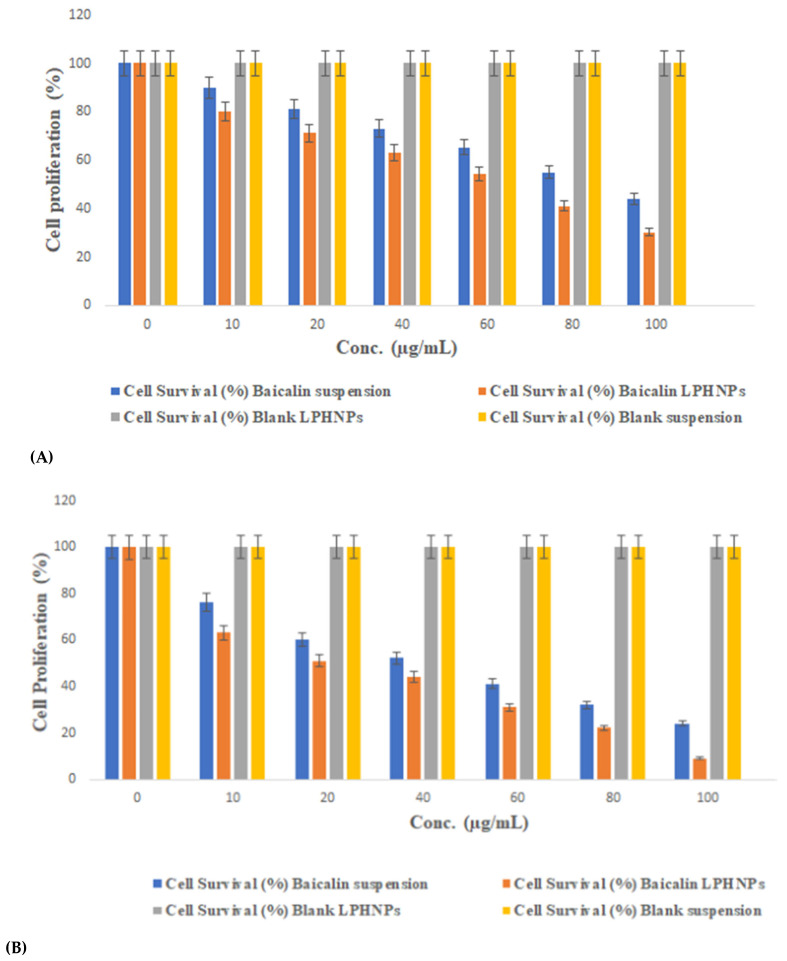
Evaluation of baicalin suspension, baicalin LPHNP, and blank LPHNP at various levels after the incubation of HCT-116 cells for (**A**) 24 h and (**B**) 48 h (LPHNPs; lipid polymeric hybrid nanoparticles). All the values were presented as mean ± SD of three independent experiments. One-way ANOVA was used for the statistical analysis. The data showed significant (*p* < 0.001) cytotoxicity compared with BQ-loaded suspension.

**Figure 6:**
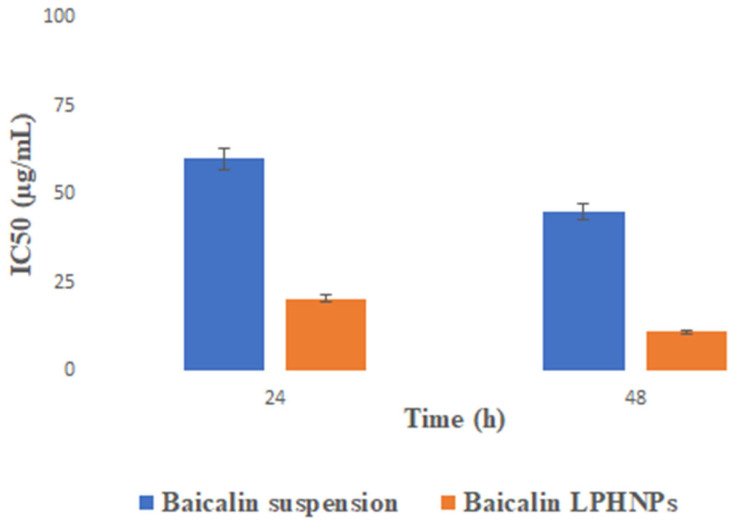
IC_50_ of baicalin suspension and baicalin-loaded LPHNPs. The data showed significant (*p* < 0.05) cytotoxicity compared to BQ-loaded suspension, after 24–48 h. All data expressed as mean ± SD (n = 3). Blank suspension and blank LPHNPs observed no toxicity in the duration of 24–48 h up to a concentration of 100 µg/mL. IC_50 =_ half maximal inhibitory concentration.

Human colon cancer HCT-116 cell lines were used to test baicalin′s cytotoxic effect on cell growth in vitro [24]. The MTT assay showed that baicalin-loaded LPHNPs, blank LPHNPs, and baicalin were all cytotoxic to HCT-116 treated for 24 and 48 h (Figure 5A,B). Half-maximal inhibitory concentration (IC_50_) values for baicalin in HCT-116 cells show a significant growth suppression (Figure 5A,B and Figure 6). Accordingly, it was shown that baicalin-loaded LPHNPs significantly decreased the viability of the HCT-116 cell line within 24 h, in comparison to baicalin suspension alone. The least viable population of baicalin-loaded LPHNPs was detected at a lower IC_50_ value after 48 h (as shown in Figure 5B and Figure 6), whereas no cytotoxicity was observed by blank suspension and blank LPHNPs up to the concentration of 100 µg/mL in the duration of 24–48 h (as shown in Figure 5A,B and Figure 6).

### 3.6. Pharmacokinetic and Organ Distribution Studies 

The in vivo drug absorption and organ distribution of the improved formulation was analyzed to assess its potential for colon targeting. 

#### 3.6.1. In Vivo Drug-Absorption Study

The pharmacokinetic parameters are shown in Table 3**.** There were significant differences in the plasma drug concentration patterns between the two formulations. For the baicalin-loaded LPHNPs, the mean peak plasma concentration (C_max_) was 12.23 ± 2.65 µg/mL, whereas for the baicalin-loaded suspension, it was 85.46 ± 6.47 µg/mL. Time to attain C_max_, i.e., T_max_ was 14.34 ± 3.05 h for baicalin-loaded LPHNPs, which was substantially greater than for baicalin suspension 1.45 ± 0.35. For LPHNPs, the MRT was 11.25 ± 2.73 h, which is 5.23 times higher than the MRT for the baicalin suspension (2.15 ± 0.25 h). For the aforementioned two formulations, the differences in the pharmacokinetic parameters (C_max_, T_max_, and MRT) were found to be significant (*p* < 0.001). This study concludes that baicalin was well-absorbed from the suspension into the systemic circulation. However, baicalin was less absorbed from the characteristic nanoparticles into the systemic circulation where the remaining fraction of the administered dose could have been expected to be retained locally in the intestinal segment. The organ distribution study data further supported the claim.

#### 3.6.2. Organ Distribution Study

**Figure 7:**
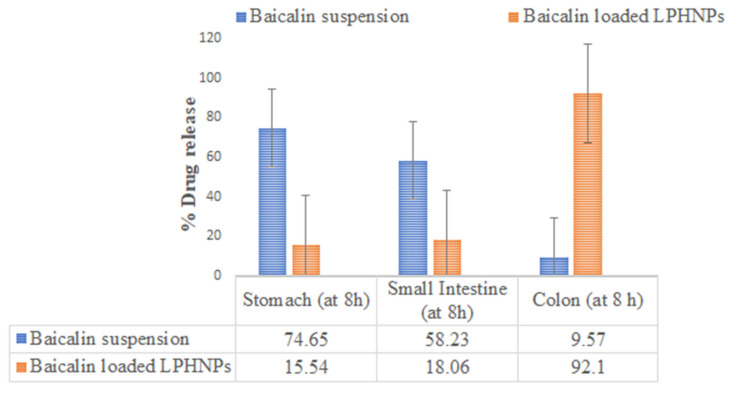
Baicalin content in GIT-isolated organs of rat after oral administration of baicalin suspension and baicalin-loaded LPHNPs. Each value represents mean ± S.D of three determinations. *p* < 0.05, compared with baicalin suspension.

The organ distribution results showed that after 8 h of oral administration of baicalin-loaded LPHNPs and baicalin suspension, the highest concentration (92.1 ± 8.1% and 9.57 ± 1.01%, respectively) of the drug was available in the colon, and least drug was available in the small intestine and stomach by LPHNPs, while maximum concentration of baicalin was released from the suspension into the stomach followed by into the intestine, and after that, a very minute amount of the drug was found in the colon from the baicalin suspension (11.34 ± 1.21) (as shown in Figure 7). Therefore, the results conclude that the maximum fraction of baicalin from LPHNPs was able to reach the colon. The in vivo experimental results, thus, were found to be inconsonant with the in vitro drug release study. 

### 3.7. Biochemical Investigation

Table 4 shows the biochemical characteristics. In comparison to the disease group, the treated group, especially the baicalin-loaded LPHNPs, most significantly reverse the level of increased biochemical parameters, including SGOT, SGPT, alkaline phosphatase, serum creatinine, and serum albumin, reaching nearest to the control group of rats over the baicalin suspension (*p* < 0.001), whereas platelets and WBC in the disease-treated group enhanced the level most effectively and reached near to the control group of rats by administrating baicalin-loaded LPHNPs. Both platelets and white blood cell counts, as well as serum creatinine and albumin levels, were significantly different between the treated groups of animals (*p* < 0.001).

## 4. Discussion

Colon cancer (CRC) is the second most common kind of cancer worldwide and the second leading cause of cancer-related death in the United States [2,3]. CRC therapies include surgery, radiation, and chemotherapy [3]. There is a critical need for alternative treatments like herbal medicine because of the prevalence of occurrences and the complexity of their underlying causes. Moreover, half of all effective medications have a natural origin; for cancer, that number reaches 60% [5]. Traditional herbal therapy has made significant contributions in the treatment of colorectal cancer, and other new components with potential anticancer properties are anticipated to be discovered in plant sources. *Scutellaria baicalensis* is widely used as a medicine in traditional Chinese medicine [9]. The anticancer properties of *S. baicalensis* are attributed to baicalin and its various chemical components have been described [9]. Baicalin, also known as 5, 6-dihydroxy-7-O-glucuronide flavone, has been demonstrated to have a wide range of pharmacological actions [9]. These include antioxidant, antiviral, anti-inflammatory, anti-HIV, and antiproliferative properties [8,9]. Intestinal microbiota metabolism following oral consumption may alter the structure and biological activity of the parent drug [8,9]. This study was undertaken with the goal of minimizing systemic absorption. We developed and studied a lipid–polymer hybrid nanoparticle delivery system for baicalin that minimizes drug release in the harsh physiological environment of the stomach and small intestine, while enhancing drug release in the colon. Baicalin-loaded LPHNPs were developed with colon targeting in consideration to deliver the drug at the most effective concentration directly to the colon. In addition, since the colon is not an absorption site for most medications, the drug produced there was not absorbed systemically. The developed baicalin-loaded LPHNPs exhibit a relatively uniform particle size distribution (PDI = 0.177) and a particle size of 184.3 nm at the 95% confidence interval. Furthermore, these results confirmed the nanometer scale of LPHNPs with homogeneous dispersion. The zeta potential exists at −19.8 mV; it revealed stable and non-aggregated formulation. The physical stability of the formulation is improved and the repulsion between the nanoparticles is increased because of the negative charge on the formulation. Additionally, the fatty acids released during the slow hydrolysis of lipids may be responsible for the negative charge [14,20], whereas the TEM photomicrograph of baicalin-loaded LPHNPs showed sizes between 160 and 200 nm. Additionally, this variance in the value of TEM as compared to the DLS method as a result of the formulation size is established by TEM in the solid state while the hydrodynamic diameter is estimated by a zeta sizer in the fluid state [21,22]. Baicalin-loaded LPHNPs and baicalin suspension with rat cecal content had a percentage release of 91.24 ± 10.11% and 9.33 ± 1.05% at the end of 8 h, whereas at the end of 24 h, for baicalin-loaded LPHNPs and baicalin suspension, their drug release was at 97.24 ± 8.11% and 11.56 ± 1.21%, respectively. The data suggested that the baicalin-loaded LPHNPs released a low quantity of drug in the stomach and intestine. The small release of cytotoxic baicalin in the stomach and small intestine suggested that LPHNPs produced from lipids and polymers hold great promise and restrict the drug release into the same. The optimized LPHNPs were shown to be stable for six weeks at all 25 °C/60% RH, but the LPHNPs remained stable at 25 °C (for 12 weeks) and at 40 °C drug loss was significant. In these findings, it is shown that the improved LPHNPs may be maintained at 25 °C/60% RH without needing to be refrigerated. Furthermore, the drug content loss was nonsignificantly varied at 25 °C/60% RH. Therefore, it concludes an excellent stability under long-term storage conditions. However up to 48 h, the baicalin-loaded LPHNPs observed the least viable population detected at a lower IC_50_ in a dose-dependent manner by baicalin-loaded LPHNPs, and no cytotoxicity was observed with blank LPHNPs and blank suspension up to 100 µg/mL for 24–48 h. The formulation was studied for its ability to target the colon by evaluating its absorption and distribution in the rat. Baicalin suspensions have higher C_max_ that can be attributed to the drug′s rapid absorption in the upper GI tract. LPHNPs formed from a mixture of lipid and polymer efficiently inhibit drug release in the upper section of the GI tract, as evidenced by the extended T_max_ observed in the case of LPHNPs formulation. Colonic microflora enzymes require around 24 h to completely degrade to lipids and polymers [17]. The low systemic toxicity of LPHNPs can be explained by their longer T_max_ and lower C_max_, meaning a lower amount of the drug is accessible systemically to interact with non-target sites. Because of their greater MRT, LPHNPs appear to have been absorbed by the colonic mucosa more gradually and steadily than conventional suspension. Baicalin was released from LPHNPs only after reaching the colon because of microbial degradation with microbial flora residing in the colon. Those findings line up with those of prior studies [14,17]. The organ distribution results showed that after 8 h of oral administration of baicalin-loaded LPHNPs and baicalin suspension, the highest concentration reaching 92.1 ± 8.1% and 9.57 ± 1.01% of the drug was available in the colon, and the least drug was made available in the small intestine and stomach by LPHNPs and the maximum amount of baicalin released from suspension into the stomach followed by into the intestine. After that, a very minute amount of drug was found in the colon in the case of the baicalin suspension. Therefore, these results demonstrate that baicalin was not released at the maximum extent from LPHNPs until they reached the colon, when they were broken down by the microbes and enzymes in the colonic microflora [17]. Furthermore, this experiment’s result is matched and supported by the in vitro and in vivo drug absorption study and observed significant statistical differences (*p* < 0.05). Therefore, it is concluded that baicalin is available to the colon at a greater extent over the baicalin-loaded suspension. In addition, the biochemical characteristics, compared with the disease group, in the treated group, especially the baicalin-loaded LPHNPs most significantly reverse the level of increased biochemical parameters, including SGOT, SGPT, alkaline phosphatase, serum creatinine, and serum albumin, reaching nearest to the control group of rats over the baicalin suspension (*p* < 0.001), whereas platelets and WBC in the disease-treated group resulted in an enhancement of the level most effectively and reached near the control group of rats by administrating baicalin-loaded LPHNPs. Therefore, this study indicated that the colon-specific formulation was successful in delivering the drug to the colon region with relatively low systemic toxicity.

## 5. Conclusions

This study created an innovative oral medication delivery system using colon-targeted LPHNPs loaded with baicalin. The experimental results showed uniform particle-sized, stable, non-aggregated formulation with higher entrapment efficiency. Baicalin-loaded LPHNPs with rat cecal content had the highest percentage of drug release at the end of 8 h. Furthermore, the formulation kept at 25 °C/60% RH for 3 months concluded monomodal particle size distribution and showed no statistically significant changes in particle size, PDI, EE, and drug contents. The least viable population of baicalin-loaded LPHNPs was detected at a lower IC_50_ value, and no cytotoxicity was observed by blank suspension and blank LPHNPs up to the concentration of 100 µg/mL. Moreover, baicalin from LPHNPs is much less absorbed and is least available in the blood plasma over the suspension. Therefore, it concludes that baicalin LPHNPs are available to the colon at greater extent over the baicalin-loaded suspension. The organ distribution results showed that after 8 h of oral administration of baicalin-loaded LPHNPs, the maximum drug was available in the colon. Whereas with the biochemical parameters, baicalin-loaded LPHNPs most significantly reverse the increased or lower value of biochemical parameters nearest to the control group of rats over the baicalin suspension. To investigate the security and effectiveness of this innovative yet useful idea, clinical investigations in humans are needed. 

## Figures and Tables

**Table 1:** Stability studies of baicalin-loaded LPHNPs following storage at 25 °C/60% RH and 40 °C/75% RH.

Months	Particle Size (nm)		PDI		EE (%)	
	Baicalin-Loaded LPHNPs at 25 °C/60% RH	Baicalin-Loaded LPHNPs at 40 °C/75% RH	Baicalin-Loaded LPHNPs at 25 °C/60% RH	Baicalin-Loaded LPHNPs at 40 °C/75% RH	Baicalin-Loaded LPHNPs at 25 °C/60% RH	Baicalin-Loaded LPHNPs at 40 °C/75% RH
0	184.3 ± 8.5	184.3 ± 12.2	0.177 ± 0.04	0.177 ± 0.03	90.12 ± 11.77	90.12 ± 8.53
1	184.5 ± 7.4	185.8 ± 8.3	0.179 ± 0.02	0.231 ± 0.05	90.10 ± 7.43	88.04 ± 6.11
2	184.9 ± 9.2	187.2 ± 12.3	0.184 ± 0.05	0.322 ± 0.06	90.05 ± 8.34	86.22 ± 6.03
3	185.1 ± 8.2	190.1 ± 9.3	0.191 ± 0.02	0.451 ± 0.08	89.98 ± 6.45	83.77 ± 4.21

PDI: polydispersity index; EE: entrapment efficiency; LPHNPs: lipid–polymer hybrid nanoparticles. All data expressed as mean ± SD (n = 3).

**Table 2:** Measurement of drug content of baicalin-loaded LPHNPs at different temperatures (n = 6).

S. No.	Weeks	% Drug Content Remaining	
		25 °C/60% RH	40 °C/75% RH
1	1	99.91 ± 2.23	99.72 ± 2.23
2	2	99.73 ± 3.43	99.24 ± 3.41
3	4	99.61 ± 3.52	98.23 ± 4.41
4	8	99.51 ± 2.73	97.45 ± 3.51
5	12	99.43 ± 3.51	96.31 ± 4.41
6	24	99.22 ± 4.61	95.45 ± 4.87
Net Loss		0.78	4.55

All data expressed as mean ± SD (n = 6).

**Table 3:** Pharmacokinetic parameters of baicalin-loaded LPHNPs and baicalin suspension after oral administration in rats.

Parameter	Baicalin-Loaded LPHNPs	Baicalin-Loaded Suspension
C_max_ (µg/mL)	12.23 ± 2.65	85.46 ± 6.47
T_max_ (h)	14.34 ± 3.05	1.45 ± 0.35
AUC0→∞ (µg h/L)	9500 ± 18.5 ^a^	20100 ± 25.23
MRT	11.25 ± 2.73	2.15 ± 0.25

Each value represents mean ± S.D of three determinations. ^a^ *p* < 0.001, compared with oral baicalin suspension.

**Table 4:** Biochemical parameters evaluated in rats treated with baicalin colon-targeted LPHNPs over baicalin suspension.

Parameters	Normal Control Group	Disease Group	Treated Group with Baicalin-Loaded LPHNPs	Treated Group with Baicalin-Loaded Suspension
SGOT (IU/L)	31.4 ± 6.23	100.2 ± 15.21	33.5 ± 4.23	70.2 ± 12.22
SGPT (IU/L)	25.8 ± 3.32	116.1 ± 11.23	26.8 ± 2.11	95.2 ± 11.14
Alkaline phosphatase (IU/L)	83.31 ± 8.23	202.2 ± 17.21	85.21 ± 7.23	135.2 ± 14.21
Serum creatinine (mg/dL)	0.47 ± 0.06	4.31 ± 0.23	0.59 ± 0.07	1.42 ± 0.12
Serum albumin (g/dL)	3.01 ± 0.07	9.11 ± 0.34	3.25 ± 0.05	6.23 ± 0.21
Platelets (Lacs/mm^3^)	3.25 ± 0.75	0.55 ± 0.06	3.81 ± 0.84	1.21 ± 0.03
WBC (/mm^3^)	6789 ± 215	1877 ± 44.21	6855 ± 250	2528 ± 74

Each value represents the (mean ± S.D) of three determinations.

## Data Availability

The authors confirm that the data supporting the findings of this study are available within the articles and can be shared upon request.
